# Effects of exercise regimens on balance ability in older patients with osteoporosis: a systematic review and Bayesian network meta-analysis of randomized controlled trials

**DOI:** 10.3389/fphys.2026.1793389

**Published:** 2026-03-31

**Authors:** Xiangyue Liu, Mengjing Chang, Huiyun Yuan, Xuemei Zheng, Wenling Tian, Dongwen Li, Dongfa Liao, Lin Cui

**Affiliations:** 1 College of Nursing, Chengdu Medical College, Chengdu, Sichuan, China; 2 Orthopaedics Department, The General Hospital of the Western Theater Command of the People’s Liberation Army, Chengdu, Sichuan, China; 3 College of Nursing, North Sichuan Medical College, Nanchong, Sichuan, China; 4 Stomatology Department, The General Hospital of the Western Theater Command of the People’s Liberation Army, Chengdu, Sichuan, China; 5 Nursing Department, The General Hospital of the Western Theater Command of the People’s Liberation Army, Chengdu, Sichuan, China

**Keywords:** balance, exercise regimen, network meta-analysis, older, osteoporosis

## Abstract

To determine the optimal exercise regimen for improving balance and preventing severe falls in older patients with osteoporosis (OP). Four databases were searched until September 1, 2025. The risk of bias was assessed using Cochrane ROB2. Outcomes included Berg Balance Scale (BBS, primary), timed up and go test (TUG), bone mineral density (BMD), one-leg stand test (OLS), and number of falls. A Bayesian network meta-analysis in R4.4.1/GeMTC synthesized effects, presented as MD (95% CrI) and ranked by SUCRA. Analysis of 22 RCTs (n=1538) versus usual care showed virtual reality (VR) most effective for BBS (MD 9.2, 95% CrI 7.2, 11; SUCRA 99.66%) and TUG (MD -4.6, 95% CrI -5.8, -3.3; SUCRA 98.51%); balance training+resistance training+aerobics (BT + RT + aerobics) best improved BMD (MD 0.016, 95% CrI 0.012, 0.020; SUCRA 72.38%); trampoline best improved OLS (MD 8.8, 95% CrI 1.7, 5.5; SUCRA 72.38%); RT most significantly reduced falls (MD 0.29, 95% CrI 0.100, 0.68; SUCRA 84.86%). For older OP patients, VR is most effective in improving overall balance and mobility, with reliable evidence. Combination training, trampoline, and RT exhibit potential benefits for BMD, OLS, and fall prevention, respectively. Due to the limited amount of evidence and network strength, however, these interventions cannot yet be considered definitive clinical recommendations. More high-quality direct comparisons are required for further validation in the future.

## 1 Introduction

Osteoporosis (OP) is a systemic metabolic disease featured with reduced bone mass and bone microarchitecture abnormalities, and it is prevalent in the older group (World congress on osteoporosis, osteoarthritis and musculoskeletal diseases (WCO-IOF-ESCEO 2025), 2025). As global aging accelerates, the prevalence of OP keeps rising, making it a significant public health issue (Wang and Hu, 2023). OP not only increases bone fragility but is also frequently accompanied by muscle atrophy, diminished neuromuscular control, and reduced proprioception, impairing patients’ balance ability (Paramento et al., 2025). Balance impairment is a primary cause of falls in older adults (Gaspar and Lapão, 2021), and the resulting fractures lead to high disability and mortality, and substantial socioeconomic burdens in older OP patients (Chen et al., 2025; The global, regional, and national burden attributab le to low bone mineral density, 1990-2020: an analysis of a modifiable risk factor from the Global Burden of Disease Study 2021, 2025). Research suggests that interventions in balance ability can improve balance ability and reduce falls in older OP patients (Paramento et al., 2025). Therefore, it is of clinical significance to identify effective strategies for enhancing balance ability and preventing falls in this group.

Exercise therapy (Sahni and Nieves, 2019), neuromuscular taping techniques (Paramento et al., 2025), and medication (Prasad et al., 2025) are available for enhancing balance ability in older OP patients nowadays. Research data on the long-term efficacy of neuromuscular taping techniques remains lacking, with insufficient strength of evidence (Paramento et al., 2025). Medication can directly modulate bone metabolism and synergize with other approaches to reduce the incidence of falls and fractures (Brooke-Wavell et al., 2022). However, long-term medication may carry adverse effects such as hepatic and renal burden, and increase economic burden. Moreover, drugs can enhance bone mineral density (BMD), but they are less effective in improving balance ability, muscle strength, and function, and thus cannot directly reduce falls (Giangregorio and Ponzano, 2022). Exercise therapy is considered a core non-pharmacological approach due to its multi-targeted effects, high safety profile, and good cost-effectiveness (Brooke-Wavell et al., 2022; Zhang et al., 2022; Gao et al., 2023; Tao et al., 2024). Evidence suggests that exercise training, such as robot-assisted or virtual reality (VR) balance training (BT), can effectively increase the Berg Balance Scale (BBS) score (Krohn et al., 2024; Hao et al., 2025) and shorten the timed up and go test (TUG) time by enhancing lower limb muscle strength and coordination (Gil et al., 2021). Furthermore, exercise positively modulates bone metabolism and improves BMD by mechanical loading and secretion of myogenic factors (e.g., irisin), and also directly enhances neuromuscular control and static one-leg stand ability (Wu et al., 2023; Hu et al., 2024; Xiong et al., 2025). With these changes in the skeletal, muscular, and nervous systems, older OP patients ultimately experience enhanced overall postural stability and balance, effectively reducing falls (Vonstad et al., 2022).

Despite substantial evidence, available studies have key limitations: The relative efficacy in ameliorating the aforementioned outcome metrics remains unclear across exercise regimens. It is difficult for traditional reviews and meta-analyses to quantitatively rank and optimize exercise regimens in the absence of head-to-head comparisons. To address these issues, we conducted this systematic review and Bayesian network meta-analysis (NMA). It directly compared the relative effects of different exercise regimens on balance ability (e.g., BBS, TUG), BMD, and falls in older OP patients, and ranked the effects of interventions by synthesizing and quantifying available evidence of randomized controlled trials (RCTs). The findings are expected to provide a high-level, evidence-based basis for developing the optimal individualized exercise regimens in clinical practice.

## 2 Materials and methods

An NMA was conducted following the PRISMA guidelines. It was registered with PROSPERO (https://www.crd.york.ac.uk/prospero/) (registration No. CRD420251158469).

### 2.1 Search strategy

We searched Embase, Cochrane, PubMed, and Web of Science from inception until September 1, 2025, with primary search terms “Osteoporosis”, “Exercise”, “Balance”, and “Older adults” (Appendix 1). No language restrictions were applied, but the English abstract should be obtained for non-English-language articles.

### 2.2 Study screening

After duplicate publications were removed using EndNote 21, two reviewers (Liu XY and Chang MJ) independently examined the title and abstract to identify relevant studies. Then the full text was downloaded and screened independently by the two reviewers, and the results were summarized by a third reviewer. The included studies comprised older OP patients, and interventions included trampoline, pulsed electromagnetic fields (PEMF), resistance training (RT), BT, aerobics, aquatic exercise (AE), VR, whole body vibration exercise (WBV), BT + RT + aerobics, BT + RT, BT + cognitive tasks (CT), BT + WBV, BT + aerobics, and usual care (Control). The outcomes were the BBS score, TUG, BMD, OLS, and number of falls. RCTs that at least met the criteria for patient characteristics and interventions were included.

### 2.3 Outcome metrics

The BBS is a core tool for clinically assessing comprehensive balance function, widely used for fall risk assessment in older stroke and Parkinson’s disease patients undergoing neurological rehabilitation. It comprises 14 items, each scored 0–4 points, with a total score of 56 points (Downs et al., 2013). Higher scores correspond to better balance function (Zhang et al., 2024).

TUG fully assesses mobility and fall risk by measuring the time (seconds) required for subjects to rise from a chair, walk 3 meters, turn around, walk back, and sit down. Longer time suggests a higher likelihood of functional decline or disability (Gatenio-Hefling et al., 2025). TUG is simple to complete and time-efficient, making it a commonly used tool for evaluating functional mobility in older adults and patients with neurological disorders.

BMD is a crucial indicator for skeletal health, which quantifies bone strength by measuring bone mineral content per unit area or volume. Reduced BMD is a significant predictor of fracture risks (The global, regional, and national burden attributab le to low bone mineral density, 1990-2020: an analysis of a modifiable risk factor from the Global Burden of Disease Study 2021, 2025). Therefore, changes in BMD are often tracked to objectively assess the progression of skeletal diseases such as OP and the effect of medication (Banefelt et al., 2022).

OLS assesses the subject’s static balance by recording the duration of successful posture maintenance or whether falls occur (Araujo et al., 2022). It can not only rapidly evaluate balance function but also reveal asymmetry in lower limb strength and coordination in a simple way. Therefore, it is frequently used for preliminary fall risk assessment in community-dwelling older adults (Bendrik et al., 2024).

The number of falls refers to the number of individuals experiencing falls within a defined time period, commonly used for health monitoring in older groups. Tracking changes in the number of falls allows for quantitative assessment of fall risks, guidance of the development and implementation of preventive interventions, and evaluation of the effectiveness of related regimens (Chen et al., 2025).

### 2.4 Eligibility criteria

Inclusion criteria: (1) Population: older patients (≥60 years) diagnosed with OP; (2) Intervention: exercise regimens; (3) Outcomes: metrics for balance ability, such as BBS and TUG; (4) Study design: RCTs.

Exclusion criteria: (1) Non-adult or animal studies; (2) unclear or non-target populations; (3) unclear interventions or drug interventions; (4) unavailable full text or no relevant data; (5) non-RCTs.

### 2.5 Data extraction

Two reviewers (Liu XY and Chang MG) reviewed the included studies and extracted data: first author, publication year, country, sample size, age, sex, BMI, interventions, controls, number of subjects in the test and control groups, study type, outcome metrics (BBS, TUG, BMD, OLS, and number of falls), and follow-up period. The mean and standard deviation (SD) pre- and post-intervention were extracted in the test and control groups. Change-from-baseline values with SD were prioritized, but if they were not reported, MD was calculated by pre- and post-intervention measurements. When SD was unavailable, we calculated standard error, 95% confidence interval (CI), range, and interquartile range (IQR). When IQR was reported, it was used as the mean and IQR/1.135 as the SD. If the Min-Max median was reported, it was not statistically analyzed. Given different scoring scales across outcome metrics, the scoring data of outcome metrics were pooled using MD. Falls, a dichotomous variable, were analyzed using the number of falls in the test and control groups post-intervention. Notably, due to variations in follow-up durations across studies and the potential impact of baseline levels of some outcome metrics (e.g., BBS, TUG) on effect sizes, a random-effects model was incorporated in the meta-analysis to partially account for heterogeneity. However, the potential impact of different follow-up durations and baseline levels on the comparability of MD still needs to be considered during result interpretation.

### 2.6 Risk of bias assessment

The included studies were assessed for the RoB using the Cochrane ROB2 tool from the randomization process, deviations from intended interventions, missing outcome data, measurement of outcome, and selection of reported results. Each domain was rated as low risk, some concern, or high risk. Two reviewers (Liu XY and Chang MG) independently assessed study quality. Any discrepancy was settled by consultation with a third reviewer (Zheng XM).

### 2.7 Data synthesis and analysis

R4.4.1 and GeMTC were utilized for NMA modeling. All data analyses were completed based on a Bayesian random-effects model supported by the R “GeMTC” and “rjags” packages. The posterior distribution of parameters was simulated via MCMC, and the Bayesian approach was used to provide probability distributions for effect size estimates. A Bayesian NMA model was ultimately created with 25,000 iterations, 250,000 simulations, and 10-fold sampling. Consistency and inconsistency tests were carried out. When closed loops were formed in the network diagram, local inconsistencies between direct and indirect evidence were assessed by the node-splitting method. Model convergence was evaluated using Brooks-Gelman-Rubin statistics combined with trace plots and density plots. Effect sizes were calculated for MD with 95% CrI by a Bayesian random-effects model. Given that falls constitute a dichotomous variable (presence or absence), a Bayesian framework-based binomial likelihood model was utilized, and the log link function was used to create a fixed-effects NMA model. Results were presented as RR with 95% CI. Comparison results were visualized by forest plots and summary tables. An effect was considered significant when its 95% CrI did not include zero. The efficacy of interventions was ranked by the surface under the cumulative ranking curve (SUCRA) value (0-100%), with higher values indicating better efficacy. Notably, SUCRA rankings reveal the probability of each intervention being the optimal intervention rather than the absolute value of effect size, and it cannot be simply equated with clinical superiority. When CIs overlap among multiple interventions, minor differences in SUCRA may reflect sampling error rather than the superiority or inferiority of interventions. Therefore, this study prioritized effect sizes with CIs, and did not rank the interventions with overlapping CIs. The relative relationships among interventions were visualized by a network diagram, where nodes represented interventions and edges represented direct comparisons.

## 3 Results

### 3.1 Search results

**Figure 1:**
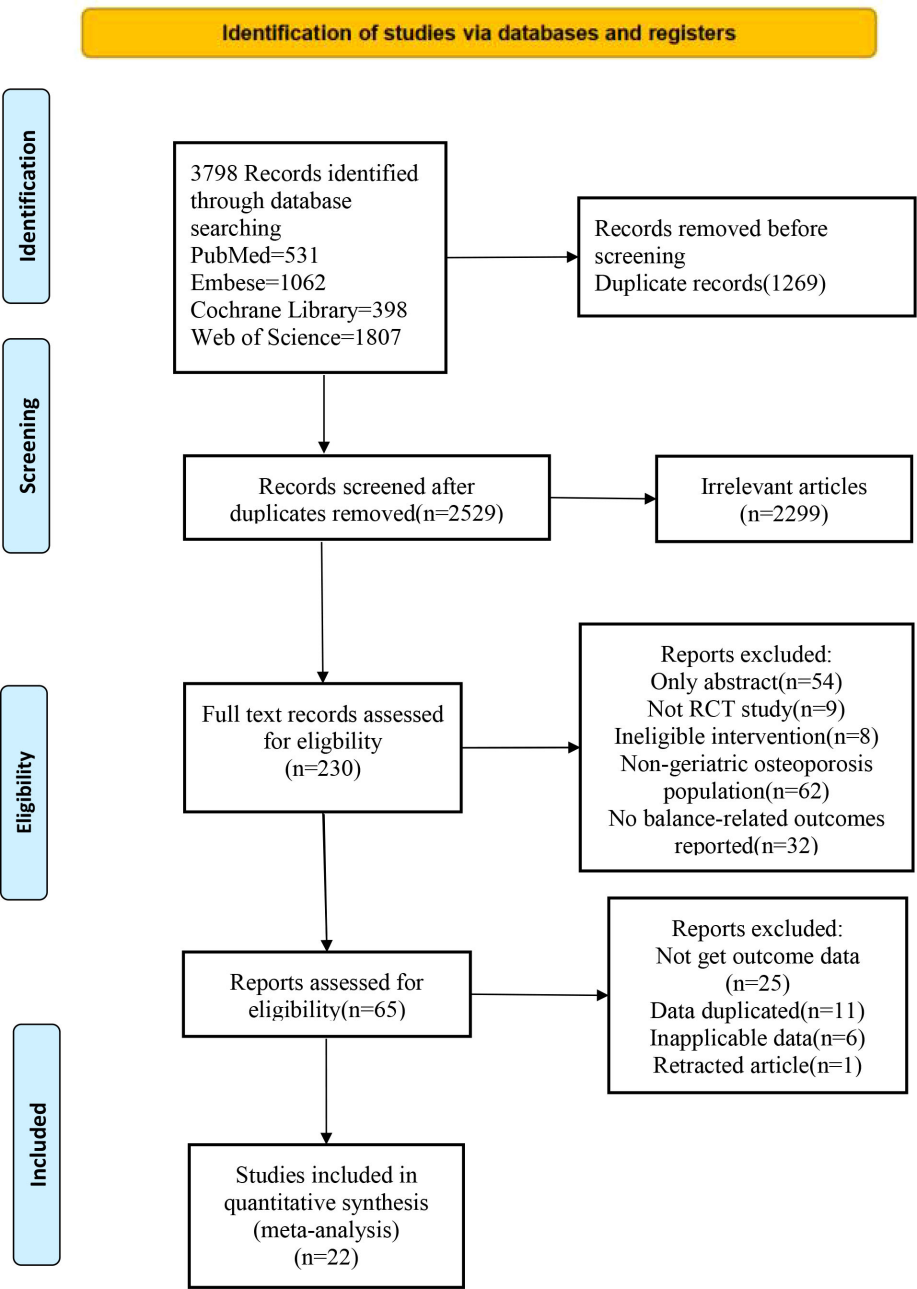
PRISMA flow diagram of the study selection process.

We initially retrieved 3798 reports, of which 1269 duplicate publications were excluded. After title and abstract review, the full text of 230 eligible studies was examined, and the ineligible studies were removed due to unavailable full text (n=54), ineligible study types (n=9), ineligible interventions (n=8), non-older OP patients (n=62), no outcome metrics for balance (n=32), no outcome data (n=25), duplicate data (n=11), non-applicable data (n=6), and withdrawn article (n=1). Finally, 22 studies were included following the PRISMA flowchart (**Figure 1**).

### 3.2 Study characteristics

Twenty-two studies (Vaillant et al., 2006; Madureira et al., 2007; Swanenburg et al., 2007; Smulders et al., 2010; Teixeira et al., 2010; Murtezani et al., 2014; Olsen and Bergland, 2014; Hakestad et al., 2015; Konak et al., 2016; Ruzene et al., 2016; Oksuz and Unal, 2017; Dizdar et al., 2018; Miko et al., 2018; Posch et al., 2019; Ramos et al., 2019; Stanghelle et al., 2020; FilipoviĆ et al., 2021; Zhang et al., 2022; Zhao et al., 2023; Mewara and Chhajed, 2024; Sangtarash et al., 2024; Yilmaz and Kösehasanoğulları, 2024) were included, involving 1538 older OP patients, and 13 interventions (trampoline, PEMF, BT, RT, AE, WBV, VR, aerobics, BT + RT, BT + aerobics, BT + RT + aerobics, BT + WBV, BT + CT) and usual care (Control). All RCTs were from Europe, South America, and Asia, and the subjects were aged ≥60 years, mostly with an excessive BMI. BMD was reported primarily in the lumbar spine, as well as the hip and femur. The follow-up duration varied greatly, ranging from immediate assessments to long-term follow-ups, with 3–6 month short- to medium-term follow-ups being most common (**Table 1**). Variations were present in training dosage across interventions. For example, BT + RT + aerobics lasted for 5.5–40 weeks, 2–3 sessions per week and 35–90 min per session, with full supervision supplemented by partial supervision. VR training lasted for 12–52 weeks, 3 sessions per week and 45–50 minutes per session, with full supervision. The specific training dosage is summarized in **Table 2**.

**Table 1:** Characteristics of included studies.

First author	Year	District	Sample	Age (mean ± sd)	Gender (female/male)	BMI	Treatment	Research type	Outcome	Follow-up period	Baseline BBS (intervention VS control)	Baseline TUG (intervention VS control)
Jacques Vaillant	2006	France	68(37/31)	73.5 ± 1.6	68/0	NA	Exercise program with concurrent cognitive tasks VS Exercise program	RCT	TUG, OLS	3 months	-	NR
M. M. Madureira	2007	Brazil	60(30/30)	73.99 ± 4.71	60/0	25.45 ± 5.00	Balance training VS Usual care	RCT	BBS, TUG	12 months	48.8 ± 4.10 VS 48.13 ± 5.36	14.31 ± 4.03 VS 13.86 ± 3.43
Jaap Swanenburg	2007	Switzerland	20(10/10)	71.2 ± 6.8	20/0	23.3 ± 3.0	Combined balance, resistance and aerobic Training VS Usual care	RCT	BBS, Number of fallers	12 months	51.40 ± 4.3 VS 53.2 ± 2.40	-
Ellen Smulders	2010	Netherlands	96(50/46)	71.0 ± 4.7	90/6	NA	NFPP VS Usual care	RCT	Number of fallers	12 months	-	-
L. E. P. P. Teixeira	2010	Brazil	100(50/50)	62.94 ± 4.68	100/0	NA	muscular strength and proprioception training program VS Usual care	RCT	BBS, TUG, Number of fallers	18 weeks	52.07 ± 3.63 VS 51.17 ± 4.10	10.74 ± 2.23 VS 11.35 ± 2.88
Ardiana Murtezani	2014	Serbia	61(31/30)	60.24 ± 6.83	61/0	41.26 ± 3.50	Aquatic exercise VS Land exercise	RCT	BMD (Lumbar vertebrae), BBS	10 months	47.29 ± 7.05 VS 46.70 ± 6.03	-
C. F. Olsen	2014	Norway	89(47/42)	71.1 ± 5.8	89/0	25.5 ± 4.2	group-based circuit exercise program VS Usual care	RCT	Number of fallers	12 months	-	-
K. A. Hakestad	2015	Norway	80(42/38)	64.74 ± 7.1	80/0	24.25 ± 3.52	Osteo Active VS Usual care	RCT	BMD (Hip total)	12 months	-	-
H. E. Konak	2016	Turkey	42(22/20)	68.33 ± 11.27	39/3	26.42 ± 7.56	Exercise program with concurrent cognitive tasks VS Exercise program	RCT	BBS, TUG, OLS	4 weeks	49.18 ± 1.86 VS 48.8 ± 1.85	12.36 ± 2.01 VS 12.7 ± 2.29
Juliana Rodrigues Soares Ruzene	2016	Brazil	29(14/15)	70.27 ± 6.59	29/0	30.20 ± 8.38	Balance training with oscillation VS Balance training without oscillation	RCT	BBS	8 weeks	53.14 ± 2.14 VS 53.47 ± 2.07	-
Sevim Oksuz	2017	Turkey	40(20/20)	60.23 ± 7.45	40/0	26.22 ± 3.42	Pilates VS Usual care	RCT	BBS, TUG	6 weeks	NR	NR
Meltem Dizdar	2018	Turkey	75(25/25/25)	60.54 ± 5.63	75/0	27.52 ± 4.89	Balance and coordination VS strengthening exercise VS aerobic	RCT	BBS, TUG	12 weeks	52.09 ± 4.4 VS 53.86 ± 1.50 VS 52.70 ± 2.60	9.43 ± 1.40 VS 8.77 ± 1.10 VS 9.35 ± 1.40
Ibolya MIKO	2018	Hungary	97(49/48)	69.31 ± 4.58	97/0	NA	Balance and aerobic exercise VS Usual care	RCT	BBS, TUG	12 months	49.23 ± 9.05 VS 48.52 ± 33.75	8.89 ± 7.38 VS 9.95 ± 12.02
Markus Posch	2019	Austria	40(20/20)	68.5 ± 6.1	40/0	24.8 ± 6.4	Mini-Trampoline training VS Usual care	RCT	TUG, OLS, BMD (Femoral neck)	12 weeks	-	5.83 ± 0.95 VS 5.45 ± 1.02
Luanda Alves Xavier Ramos	2019	Brazil	34(17/17)	70.94 ± 5.76	32/2	29.71 ± 5.96	WBV VS Walk	RCT	BBS, TUG	immediate effect	53.06 ± 2.36 VS 52.59 ± 3.64	7.64 ± 1.19 VS 8.29 ± 2.67
B. Stanghelle	2020	Norway	149(76/73)	74.2 ± 5.8	149/0	23.2 ± 3.7	Multicomponent resistance and balance exercise VS Usual care	RCT	TUG	12 weeks	-	6.50 ± 1.97 VS 6.52 ± 3.10
Tamara N. FILIPOVIĆ	2021	Serbia	96(47/49)	64.30 ± 5.24	96/0	25.80 ± 3.80	Multicomponent exercise VS Usual care	RCT	TUG, OLS	12 weeks	-	12.87 ± 2.45 VS 13.11 ± 2.51
F Zhang	2022	China	68(34/34)	68.4 ± 4.7	57/11	22.5 ± 3.4	HBRE VS Usual care	RCT	BBS	12 weeks	50.10 ± 2.50 VS 49.9 ± 2.70	-
Rui Zhao	2023	China	50(25/25)	72.76 ± 3.47	29/21	26.74 ± 5.23	VR VS Usual care	RCT	Number of fallers	12 months	-	-
Navin Mewara	2024	India	154(77/77)	72.05 ± 5.74	154/0	26.55 ± 4.34	A community-based, peer-led exercise VS Usual care	RCT	TUG, BMD (Lumbar spine), Number of fallers	6 months	-	11.80 ± 2.70 VS 12.1 ± 2.90
Fatemeh Sangtarash	2024	Iran	30(15/15)	61.77 ± 3.29	30/0	25.48 ± 1.72	PEMF VS Resistance Exercises	RCT	TUG	3 months	-	12.18 ± 2.20 VS 12.85 ± 1.55
Nihal Yilmaz	2024	Turkey	60(30/30)	67.5 ± 9.81	60/0	NA	Wii-based balance exercises VS Home balance exercise	RCT	BBS, TUG	12 weeks	42.7 ± 3.15 VS 41.93 ± 2.26	10.81 ± 1.79 VS 11.09 ± 1.55

RCT, Randomized Controlled Trial; TUG, Timed Up and Go Test; OLS, One Leg Stand; BBS, Berg Balance Scale; BMD, Bone Mineral Density; NFPP, the Nijmegen Falls Prevention Program; WBV, Whole Body Vibration Exercise; HBRE, Home-based resistance exercise; VR, Virtual Reality game; PEMF, Pulsed Electromagnetic Fields; NR, Only change scores were reported.

**Table 2:** Dose characteristics of exercise interventions.

Intervention type	Representative studies	Duration (weeks)	Frequency (sessions/week)	Session duration (minutes)	Supervision mode
Trampoline	Posch 2019	12	2	45-60	Supervised
PEMF	Sangtarash 2024	12	2	45	Supervised
BT + RT + aerobics	Swanenburg 2007; Smulders 2010; Murtezani 2014; Olsen 2014; Filipović 2021; Mewara 2024	5.5-40	2-3	35-90	Supervised/Partially supervised
RT	Teixeira 2010; Dizdar 2018; Zhang 2022; Sangtarash 2024	12-24	2-3	45-60	Supervised/Home-based
BT	Vaillant 2006; Madureira 2007; Konak 2016; Ruzene 2016; Dizdar 2018; Yilmaz 2024	4-52	1-3	30-60	Supervised/Home-based
BT + RT	Hakestad 2015; Stanghelle 2020	12-24	2-3	60	Supervised
BT + CT	Vaillant 2006; Konak 2016	4-12	2-3	45	Supervised
aerobics	Oksuz 2017; Dizdar 2018	6-12	3	30-60	Supervised
AE	Murtezani 2014	40	3	35	Supervised
VR	Zhao 2023; Yilmaz 2024	12-52	3	45-50	Supervised
WBV	Ramos 2019	Single session	1	8	Supervised
BT + WBV	Ruzene 2016	8	2	30	Supervised
BT + aerobics	Miko 2018	52	3	30	Partially supervised

1.Intervention duration, frequency, and session duration were extracted from the original studies; supervision mode was categorized as “fully supervised”, “partially supervised”, or “home-based/unsupervised”.

2.PEMF, Pulsed Electromagnetic Fields; BT, balance training; RT, resistance training; CT, cognitive tasks; AE, aquatic exercise; VR, Virtual Reality game; WBV, Whole Body Vibration Exercise.

### 3.3 RoB

**Figure 2:**
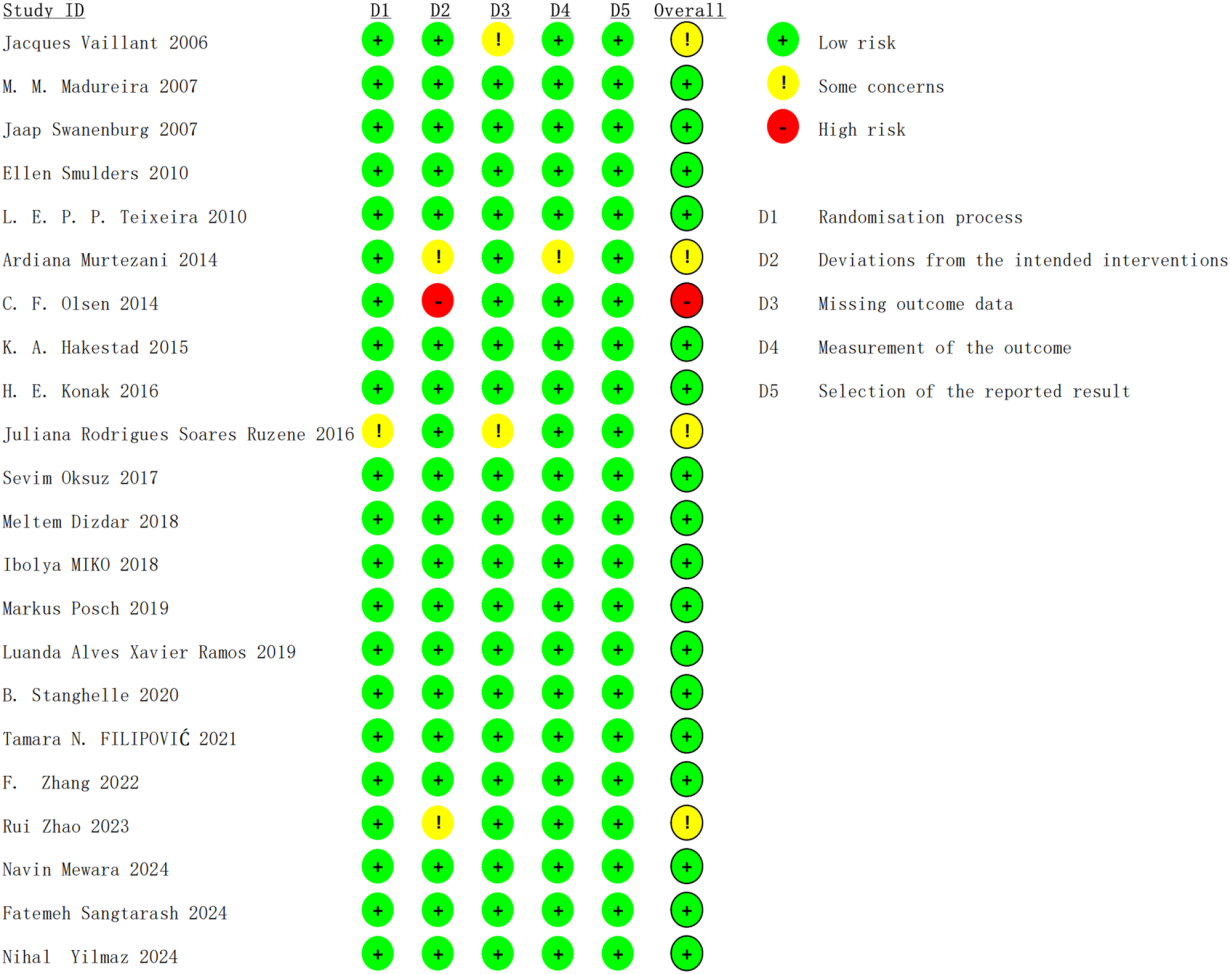
Risk of bias summary graph.

**Figure 3:**
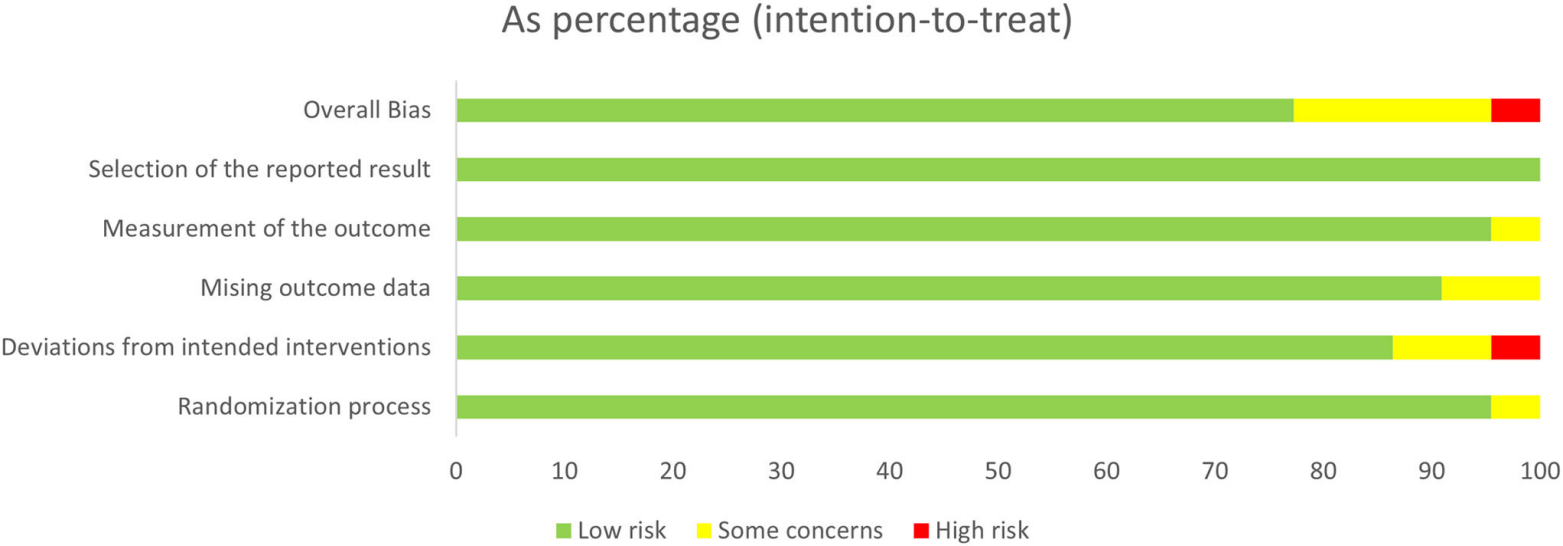
Bar chart of risk of bias assessment results.

Excel was used for data processing and chart generation for all studies (Vaillant et al., 2006; Madureira et al., 2007; Swanenburg et al., 2007; Smulders et al., 2010; Teixeira et al., 2010; Murtezani et al., 2014; Olsen and Bergland, 2014; Hakestad et al., 2015; Konak et al., 2016; Ruzene et al., 2016; Oksuz and Unal, 2017; Dizdar et al., 2018; Miko et al., 2018; Posch et al., 2019; Ramos et al., 2019; Stanghelle et al., 2020; FilipoviĆ et al., 2021; Zhang et al., 2022; Zhao et al., 2023; Mewara and Chhajed, 2024; Sangtarash et al., 2024; Yilmaz and Kösehasanoğulları, 2024). For the randomization process, one study was assessed as some concern due to no reporting of the implementation method. For the deviations from intended interventions, some studies lacked descriptions of blinding or failed to meet requirements for blinding, but they were assessed as low risk because most deviations were attributed to the study environment and did not affect outcomes or interventions. Two studies were assessed as some concern because the deviation affected outcome measurement. One study was assessed as high risk because the deviation affected outcome measurement and whether it was homogeneous between groups remained unclear. For the missing outcome data, all or nearly all outcome data were available for most studies; one study had approximately 17% follow-up attrition but performed imputation, thus rated as some concern. Another study had nearly 20% follow-up attrition, also rated as some concern. For measurement of outcome, one study was rated as some concern because the assessors might have been aware of the intervention. For the selection of reported results, all studies described the pre-specified analysis plans and were rated as low risk (**Figure 2** and **3**).

### 3.4 Assessment results of transitivity assumption

The validity of NMA relies on transitivity assumptions, i.e., clinical and methodological characteristics of studies should be comparable when conducting indirect comparisons between different interventions. To validate this assumption, the distribution of key effect modifiers across different intervention nodes was compared. Baseline information on age, BBB scores, and body mass index in intervention groups is shown in **Table 1**. The results revealed that the mean values of these key indicators were similar across intervention groups, and the ranges of individual variation reflected by SD were highly overlapped. This suggests that the patient populations in the included studies shared similar clinical characteristics, satisfying the prerequisite for transitivity. To further assess comparability of training intensity, the dosage parameters (frequency, duration, and supervision mode) of each intervention were standardized and compared (**Table 2**). The results revealed that the training intensity exhibited certain heterogeneity across nodes, but its distribution was generally balanced with no systematic bias. We considered that the integrated effect of multi-component interventions is not a simple summation of single-component interventions and should not be separated for analysis. Moreover, treating multi-component interventions as independent nodes better aligns with the pattern of intervention protocols in clinical practice. Therefore, this study incorporated both single- and multi-component interventions as independent nodes into the network. However, training intensity, specific implementation settings, supervision formats, and compliance varied across interventions, which might still influence the transitivity assumption. Besides, the node-splitting analysis revealed no statistically significant inconsistency between direct and indirect comparisons of the outcome metrics with closed loops formed (BBS and TUG) (P>0.05; **Supplementary Tables 1** and **2**). This further supported the transitivity assumption at the statistical level. Due to the extremely limited number of studies and sparse network structure for the three outcomes (BMD, OLS, and number of falls), effective subgroup analyses or meta-regression could be conducted for detailed variables such as training intensity, specific movement types, and training dosage. Consequently, the ranking results for these three outcomes should be regarded as exploratory findings, necessitating further validation by high-quality, large-sample RCTs.

### 3.5 Statistical analysis results

#### 3.5.1 Effects of different interventions on BBS scores in older OP patients

**Figure 4:**
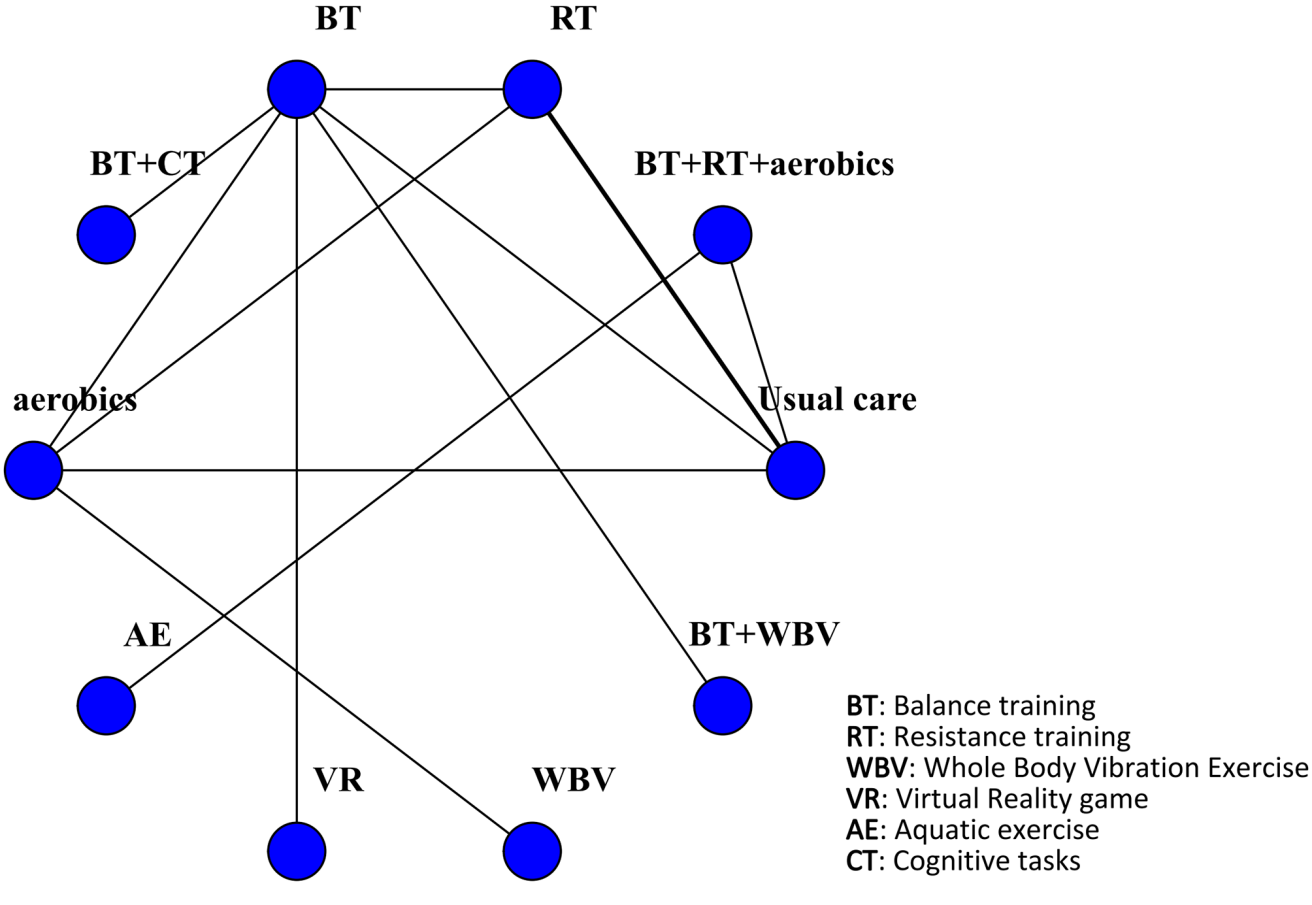
Network diagram of included studies for BBS scores.

**Figure 5:**
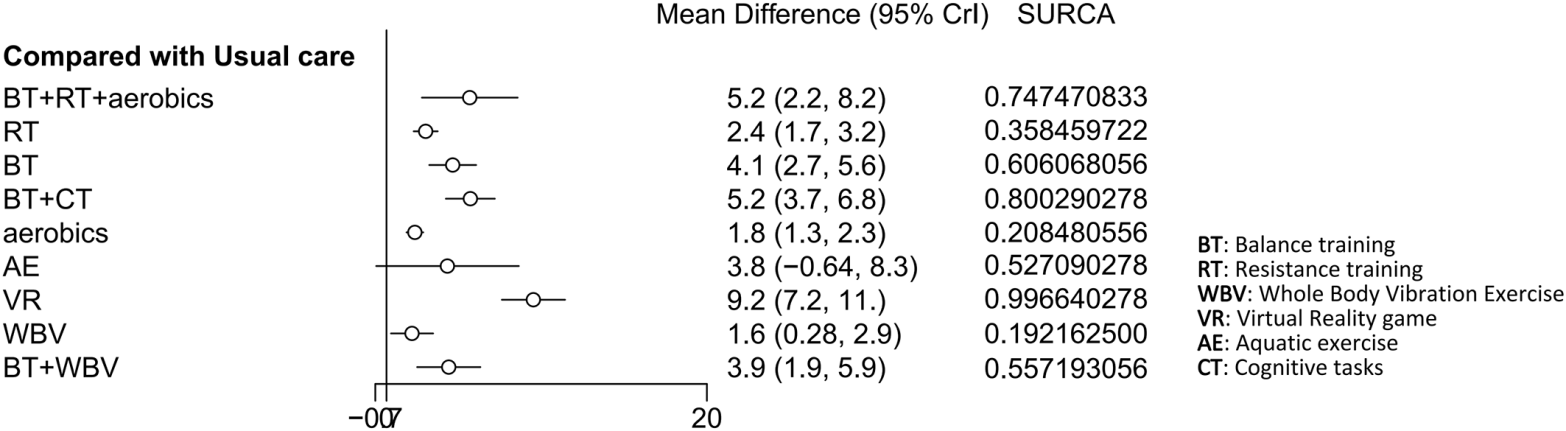
Forest plot of relative effects for BBS scores.

The BBS score was reported in 11 studies involving 574 patients. The interventions comprised BT + RT + aerobics, RT, BT, BT + CT, aerobics, AE, VR, WBV, and BT + WBV. Compared with usual care, VR was most effective in increasing the BBS score (MD 9.2, 95% CrI 7.2, 11) (SUCRA 99.66%). Additionally, BT + CT (MD 5.2, 95% CrI 3.7, 6.8) (SUCRA 80.00%). Although VR had the highest rank in terms of the SUCRA value, overlapping CIs in some comparisons suggested that the true differences in interventions might be less significant. Evidence revealed a minimal clinically important difference (MCID) of 7-11.5 points for BBS in older OP patients (Tamura et al., 2022; Kobayashi et al., 2024), and the effect size of VR fell within this range. BT + RT + aerobics (MD 5.2, 95% CrI 2.2, 8.2) (SUCRA 74.74%), BT (MD 4.1, 95% CrI 2.7, 5.6) (SUCRA 60.60%), BT + WBV (MD 3.9, 95% CrI 1.9, 5.9) (SUCRA 55.72%), RT (MD 2.4, 95% CrI 1.7, 3.2) (SUCRA 35.85%), aerobics (MD 1.8, 95% CrI 1.3, 2.3) (SUCRA 20.85%), and WBV (MD 1.6, 95% CrI 0.28, 2.9) (SUCRA 19.22%) all significantly increased the BBS score. However, their ranking differences should be interpreted carefully as the CIs of these interventions partially overlapped with VR. AE (MD 3.8, 95% CrI -0.64, 8.3) (SUCRA 52.70%) was less effective. Four closed loops were formed in the network: BT - RT - aerobics; BT - RT - usual care; BT - aerobics - usual care; RT - aerobics - usual care. The inconsistency test revealed P-values >0.05 for all comparisons of interventions, suggesting no inconsistency. The results were visualized by the network diagram (**Figure 4**), forest plot (**Figure 5**), league table (**Supplementary Figure 1**), and inconsistency test diagram (**Supplementary Table 1**; **Supplementary Figure 2**).

#### 3.5.2 Effects of different interventions on TUG time in older OP patients

**Figure 6:**
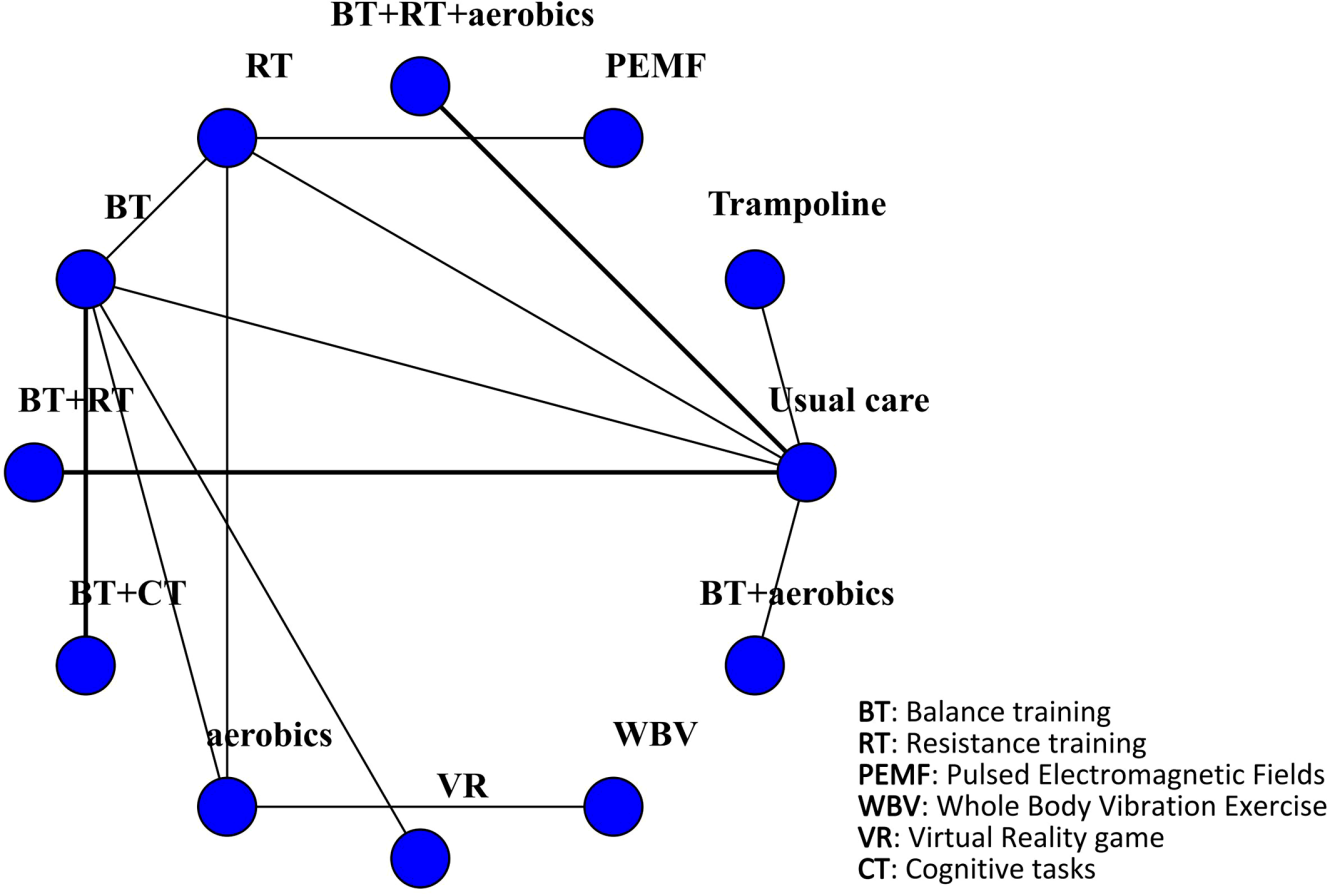
Network diagram of included studies for TUG.

**Figure 7:**
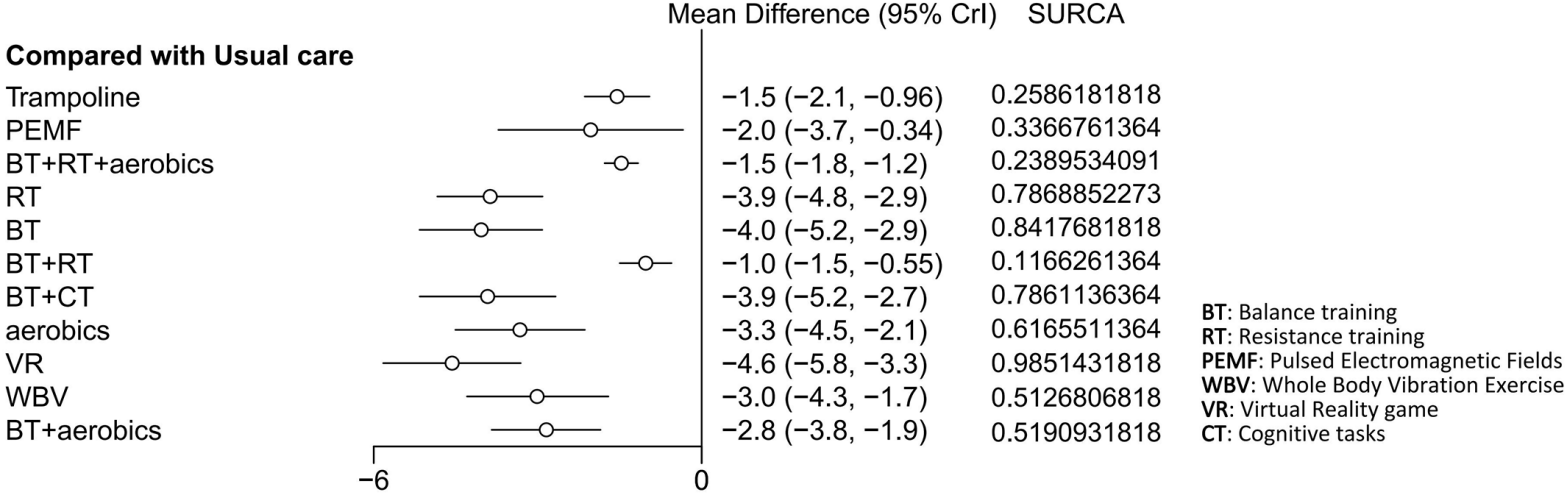
Forest plot of relative effects for TUG.

The TUG was reported in 14 studies involving 1030 patients. The interventions comprised trampoline, PEMF, BT + RT + aerobics, RT, BT, BT + RT, BT + CT, aerobics, VR, WBV, and BT + aerobics. Compared with usual care, VR was most effective in shortening TUG time (MD -4.6, 95% CrI -5.8, -3.3) (SUCRA 98.51%). Additionally, BT (MD -4.0, 95% CrI -5.2, -2.9) (SUCRA 84.18%), RT (MD -3.9, 95% CrI -4.8, -2.9) (SUCRA 78.69%), BT + CT (MD -3.9, 95% CrI -5.2, -2.7) (SUCRA 78.61%), aerobics (MD -3.3, 95% CrI -4.5, -2.1) (SUCRA 61.66%), BT + aerobics (MD -2.8, 95% CrI -3.8, -1.9) (SUCRA 51.91%), WBV (MD -3.0, 95% CrI -4.3, -1.7) (SUCRA 51.27%), PEMF (MD -2.0, 95% CrI -3.7, -0.34) (SUCRA 33.67%), trampoline (MD -1.5, 95% CrI -2.1, -0.96) (SUCRA 25.86%), BT + RT + aerobics (MD -1.5, 95% CrI -1.8, -1.2) (SUCRA 23.90%), and BT + RT (MD -1.0, 95% CrI -1.5, -0.55) (SUCRA 11.66%) all significantly shortened TUG time. However, the CIs of interventions overlapped, so the subtle differences in SUCRA rankings lacked statistical significance. Two closed loops were formed in the network: BT - RT - aerobics; BT - RT - usual care. The BT - RT - aerobics closed loop originated from a three-arm RCT, which did not establish independent direct and indirect evidence paths. Thus, the rigorous inconsistency test was precluded. The inconsistency test revealed P-values >0.05 for all comparisons of interventions, suggesting no inconsistency. The results were visualized by the network diagram (**Figure 6**), forest plot (**Figure 7**), league table (**Supplementary Figure 3**), and inconsistency test diagram (**Supplementary Table 2**; **Supplementary Figure 4**).

#### 3.5.3 Effects of different interventions on BMD in older OP patients

**Figure 8:**
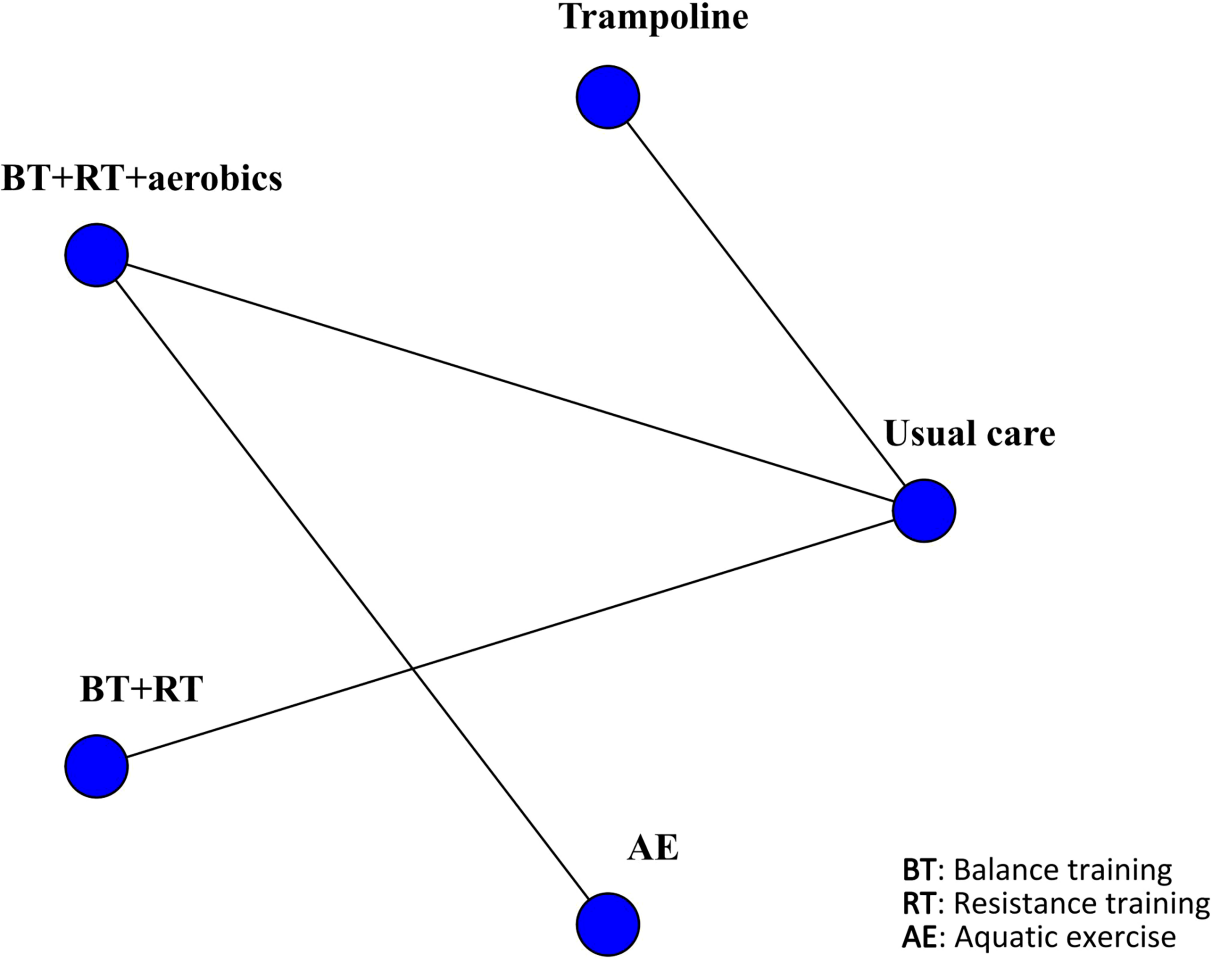
Network diagram of included studies for BMD.

**Figure 9:**
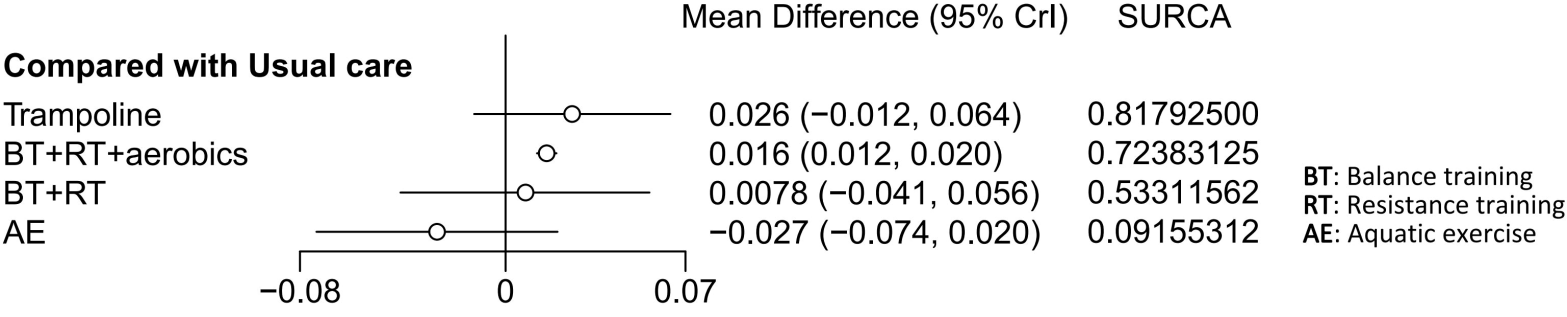
Forest plot of relative effects for BMD.

The BMD was reported in four studies involving 319 patients. The interventions included trampoline, BT + RT + aerobics, BT + RT, and AE. Compared with usual care, BT + RT + aerobics performed best in improving BMD (MD 0.016, 95% CrI 0.012, 0.020) (SUCRA 72.38%). Additionally, trampoline (MD 0.026, 95% CrI -0.012, 0.064) (SUCRA 81.79%), BT + RT (MD 0.0078, 95% CrI -0.041, 0.056) (SUCRA 53.31%), and AE (MD -0.027, 95% CrI -0.074, 0.020) (SUCRA 9.16%) did not improve BMD. Although the pooled effect size of trampoline was larger (MD 0.026), its 95% CI included invalid values (-0.012, 0.064), indicating a non-robust finding that trampoline should not be regarded as superior to BT + RT + aerobics. Only four studies were included for BMD, resulting in a sparse network structure with no closed loop formed. Consequently, the consistency test was not conducted, and the SUCRA ranking was significantly affected by sampling error. Given the limited strength of evidence, these interventions cannot yet be considered a definitive clinical recommendation. The results were visualized by the network diagram (**Figure 8**), forest plot (**Figure 9**), and league table (**Supplementary Figure 5**).

#### 3.5.4 Effects of different interventions on OLS in older OP patients

**Figure 10:**
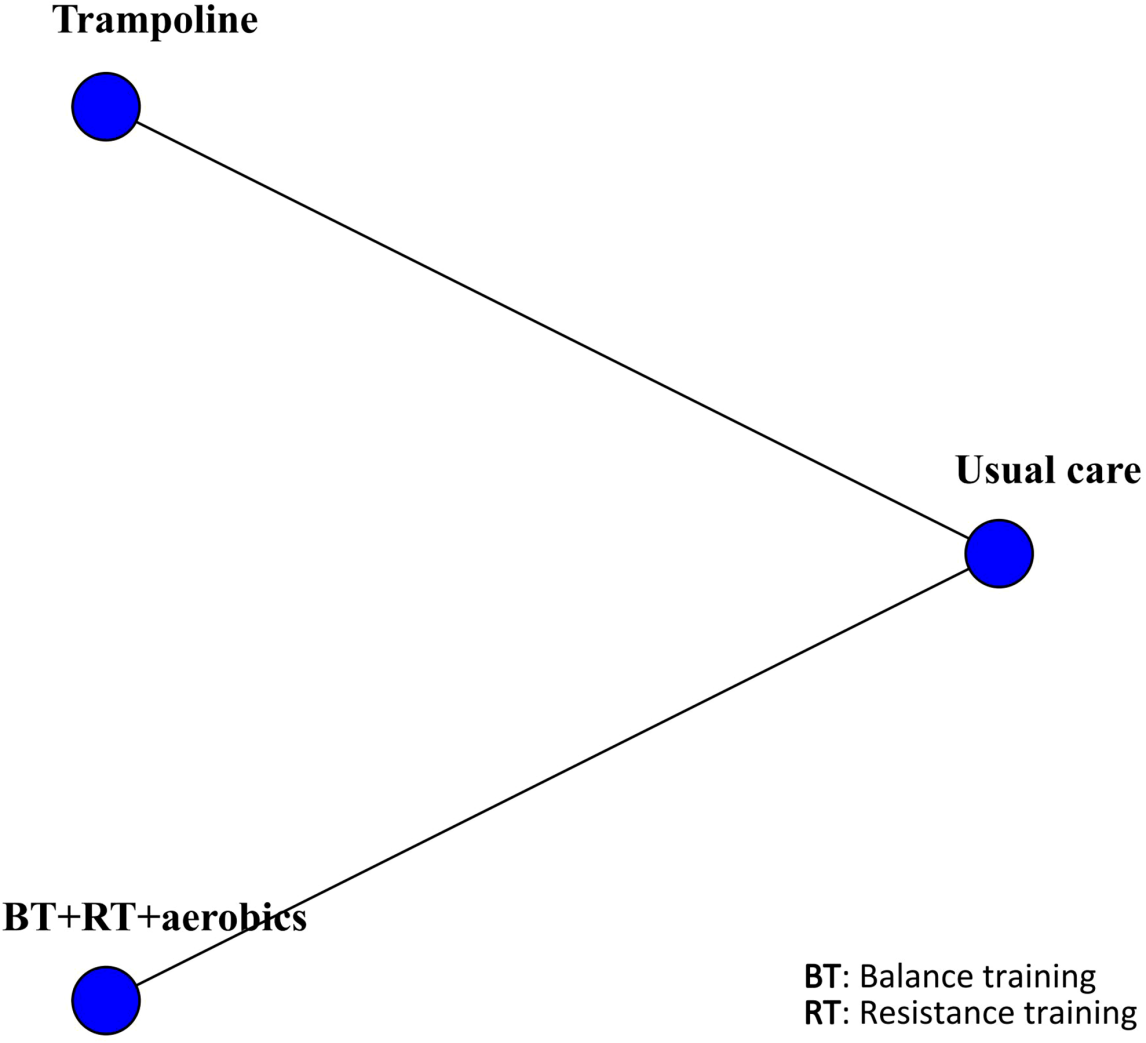
Network diagram of included studies for OLS.

**Figure 11:**
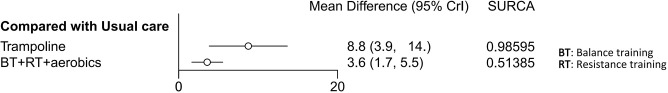
Forest plot of relative effects for OLS.

Two studies reported OLS, involving 136 patients. The interventions included trampoline and BT + RT + aerobics. Compared with usual care, trampoline performed best in improving OLS (MD 8.8, 95% CrI 3.9, 14.0) (SUCRA 98.60%). Additionally, BT + RT + aerobics also significantly regulated OLS (MD 3.6, 95% CrI 1.7, 5.5) (SUCRA 51.39%). Only two studies were included for OLS, resulting in an extremely limited network structure. Direct comparisons could only be made with usual care, precluding complex indirect comparisons. The SUCRA ranking was highly susceptible to incidental effects and should not serve as a basis for judging the superiority or inferiority of interventions. This finding is merely suggestive and far from the strength required for clinical recommendations. More RCTs are required to validate this finding. The results were visualized by the network diagram (**Figure 10**), forest plot (**Figure 11**), and league table (**Supplementary Figure 6**).

#### 3.5.5 Effects of different interventions on falls in older OP patients

**Figure 12:**
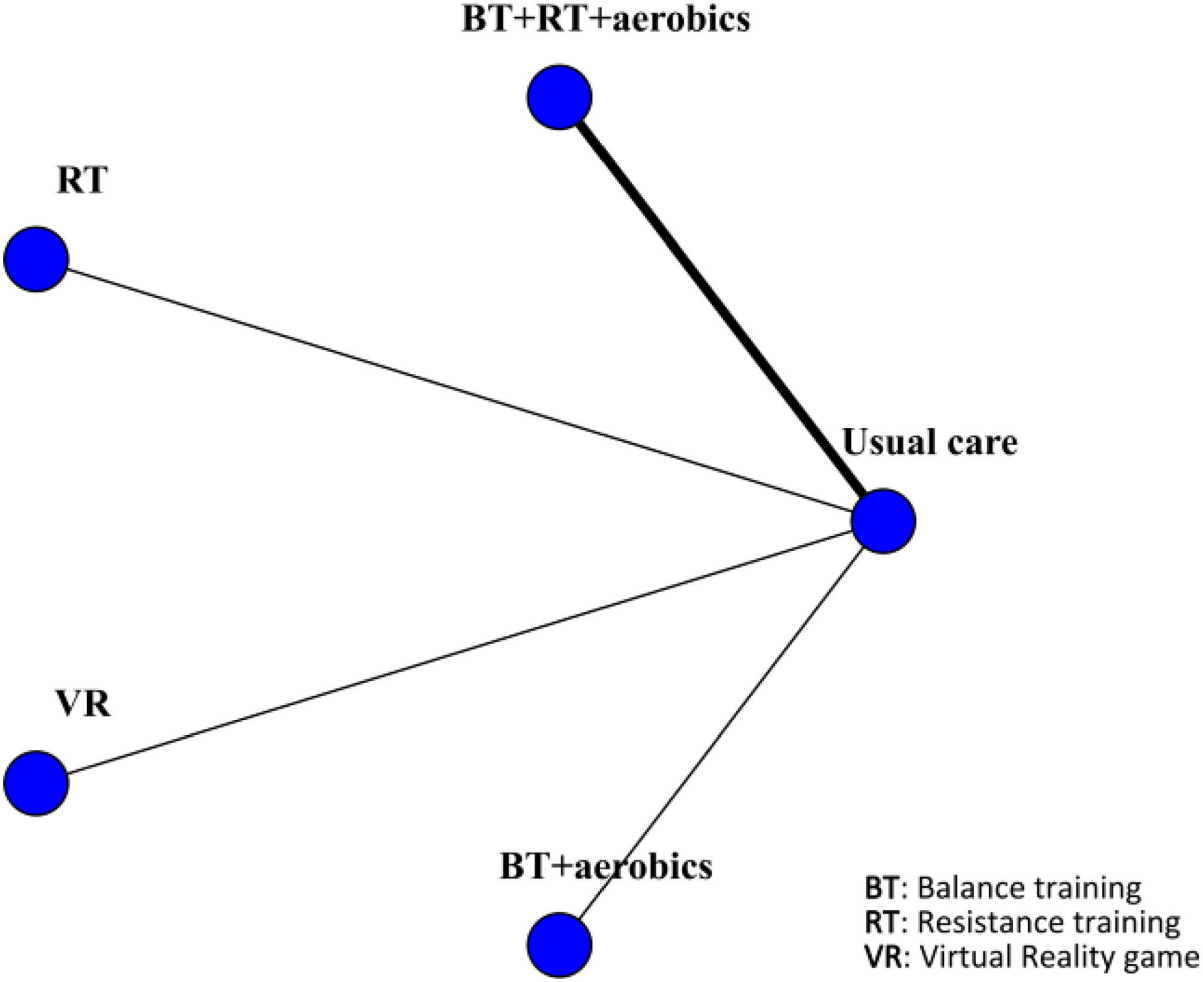
Network diagram of included studies for falls.

**Figure 13:**
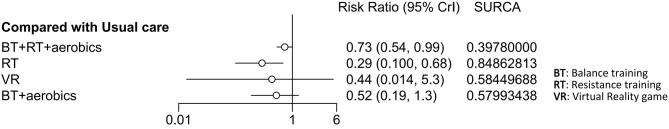
Forest plot of relative effects for falls.

Seven studies reported the incidence of falls, involving 586 patients. The interventions included BT + RT + aerobics, RT, VR, and BT + aerobics. Compared with usual care, RT was most effective in reducing falls (RR 0.29, 95% CrI 0.100, 0.68) (SUCRA 84.86%). Additionally, BT + RT + aerobics also reduced falls (RR 0.73, 95% CrI 0.54, 0.99) (SUCRA 39.78%), but VR (RR 0.44, 95% CrI 0.014, 5.3) (SUCRA 58.45%) and BT + aerobics (RR 0.52, 95% CrI 0.19, 1.3) (SUCRA 57.99%) were less effective. Although seven studies reported falls, direct comparisons of different interventions remained scarce. The network was weakly connected, sufficient indirect comparison was lacking, and no closed loop was formed, thus precluding the inconsistency test. In this case, the SUCRA ranking only reflected the model’s estimated probability rather than directly representing the definite clinical superiority of interventions. Studies exhibited heterogeneity in the definition of falls and follow-up durations. Therefore, the preventive effect of RT on falls should be regarded as preliminary evidence, requiring more high-quality studies with standardized outcome reporting. The results were visualized by the network diagram (**Figure 12**), forest plot (**Figure 13**), and league table (**Supplementary Figure 7**).

## 4 Discussion

This Bayesian NMA fully compared the effects of 13 exercise regimens on balance ability and related outcomes in older OP patients. Different exercise regimens demonstrated varying effects on outcome metrics. Specifically, VR ranked higher in improving overall balance function (BBS) and functional mobility (TUG), with effect sizes reaching the lower limit of MCID. Trampoline exhibited potential for enhancing static balance (OLS), but the strength of evidence was extremely low. BT + RT + aerobics produced a significant effect on increasing BMD. Furthermore, RT alone showed a statistically significant association with reduced fall risk. These findings offered guidance for the direction of clinical exercise prescription development. However, the strength of evidence considerably varied across outcome metrics and warrants stratified interpretation in subsequent discussions.

The BBS score was reported in 11 studies involving 574 patients, and the TUG was reported in 14 studies involving 1030 patients. The results showed that VR outperformed usual care in improving both BBS and TUG. The possible reason is that in an immersive, task-oriented environment, VR can stimulate cognitive engagement in physical activity, promote neuroplasticity, and optimize multisensory integration and neuromuscular coordination. In this way, efficient training of dynamic balance and executive function can be achieved (Barbanchon et al., 2024; Intziegianni et al., 2024). In clinical care, nurses or rehabilitation therapists can utilize VR devices to design training tasks simulating daily scenarios such as stepping over barriers or bending to pick up objects. In this way, neuromuscular control and cognitive-motor integration can be directly trained within a safe, controlled environment (Rezaei et al., 2023). Moreover, patients can embrace higher practical ability and confidence to handle complex environments by enhancing training motivation and relieving fear (Cortés-Pérez et al., 2025). Ultimately, VR improves overall function better than traditional methods (Zhao et al., 2023). In contrast, AE was less effective in improving BBS. The possible reason is that weight-bearing and balance challenges are reduced due to its buoyancy, and neuromuscular control required for walking fails to be sufficiently stimulated (Deng et al., 2024). Similarly, PEMF and some single training ameliorated TUG, but they may not be optimal choices for complex, coordinated tasks of functional mobility. From a perspective of clinical practicality, VR devices are expensive and have high requirements for service environment and technical support, thus restricting their accessibility in resource-constrained medical settings or families. Therefore, VR remains the preferred choice for balance improvement in well-equipped hospitals or rehabilitation centers. In community or primary care settings, physical functional training simulating VR principles can be implemented under the guidance of healthcare staff as a cost-effective alternative. As proposed by Lars et al., VR can be utilized for precise assessment and complex scenario training in specialized institutions, while simplified training protocol can be used in primary care settings (Donath et al., 2016). This low-cost, highly accessible human-guided training mode can be compared with VR for efficacy in future studies, and cost-effectiveness analysis can be performed. The findings are expected to providing more universally applicable protocols in clinical practice across resource contexts.

The effects of exercise therapy on bone structure (BMD) and static balance (OLS) were also specific to regimens. Four studies reported BMD, involving 319 patients. They identified BT + RT + aerobics as the optimal intervention, which worked by fully stimulating osteoblast activity under diverse, integrated mechanical loads (Hejazi et al., 2022; Altai et al., 2024). This finding aligned with a study by Borja Sañudo et al. on the mechanism of exercise intervention in OP (Sañudo et al., 2025). However, the robustness of these conclusions was restricted by the following factors: Only four studies were included, resulting in a sparse network structure, and training dosage varied across studies. Therefore, the improvement effect of combination training on BMD should be regarded as an exploratory finding. Combination training can serve as a reference for long-term bone health maintenance but requires individualized adjustment in clinical practice based on the patient’s specific circumstances. In addition, two studies reported OLS, involving 136 patients. Trampoline was identified as an effective intervention, as postural control can be continuously challenged by the unstable surface of a trampoline, thereby significantly activating core and ankle-stabilizing muscles. This is an underlying mechanism for its significant effect on static balance (Posch et al., 2019; Tay et al., 2019). Nevertheless, extreme caution is needed when applying trampoline to older OP patients. The reason is that the conclusion was based solely on two small-sample studies, with an extremely weak evidence base. OP patients face potential risks of vertebral compression fractures, falls, and joint injuries during trampoline, and insufficient safety data are available on its routine use in vulnerable populations. Therefore, trampoline should not be recommended as an independent or routine intervention and requires further exploration.

Fall incidents were evaluated in seven studies involving 586 patients. RT showed a statistically significant association with reduced fall risk, whose core mechanism lies in rapidly enhancing the absolute strength and explosive power of lower limb and core muscles (da Rosa Orssatto et al., 2019; González-Gálvez et al., 2024). It offers a critical physiological foundation for effective protective responses to unexpected loss of balance (Kitada et al., 2025). Notably, VR and BT + aerobics did not significantly prevent falls. Prevention of falls relies not only on improved balance and cognition but more on maximal muscle strength that supports rapid responses to sudden events. VR may not fully train rapid integration of vestibular sensation and proprioception, while BT + aerobics emphasizes regular movement and lacks stress training for unexpected imbalance scenarios (Sadeghi et al., 2021; Ketterer et al., 2024). As a result, they are both less effective in preventing falls. In clinical practice, RT possesses high cost-effectiveness and can greatly improve symptoms and enhance muscle strength without the need for expensive devices. Moreover, it can also be easily mastered and taught by healthcare providers while being highly adaptable for home use (Ferrari et al., 2024). Designing progressive plans and incorporating home exercises (e.g., resistance bands) should be able to effectively enhance patients’ long-term adherence. Jackson et al. also argued that regardless of the exercise regimen, sufficient progressive RT should be included (Fyfe et al., 2022). In practice, nurses can guide patients with RT as the core component and encourage their families to participate in safe home exercises under professional supervision. In this way, social and family support can be systematically integrated throughout the rehabilitation process. However, the following methodological limitations warrant close attention: First, no closed loop was formed in the network, precluding the inconsistency test. Second, heterogeneity was present in the definition of fall and follow-up durations. Therefore, the preventive effect of RT on falls should be regarded as preliminary evidence rather than a definitive conclusion. Nevertheless, based on the definite physiological basis of muscle strengthening, progressive RT is still recommended as a basic component of all exercise prescriptions. RT can be implemented by progressive home exercise to enhance accessibility.

Our findings aligned with and further expanded the available evidence. Multiple studies and guidelines recommend a multi-modal exercise regimen (Zhao et al., 2017; Brooke-Wavell et al., 2022), consistent with our conclusion that combination training is most effective in improving BMD. However, this Bayesian NMA fully quantified and ranked the efficacy of exercise regimens in older OP patients, achieving more valuable synthesis of evidence for clinical decision-making. Although this study strictly followed the PRISMA-NMA guidelines, some limitations remained. First, heterogeneity was found in exercise regimens regarding training intensity, frequency, and supervision conditions. Its impact on key outcomes (e.g., BBS, TUG) was controllable, but it may amplify bias in indirect comparison of outcomes with sparser network structures (e.g., BMD, OLS, and falls). Second, only BBS was discussed in conjunction with MCID, so whether some statistically significant intervention effects have clinical practical value remains to be verified. Moreover, the vast majority of studies had durations of interventions ≤12 months, precluding understanding of long-term effects and safety. Key indicators for scientific implementation, such as safety, cost, and acceptability, were not reported in the original studies, preventing a comprehensive assessment of the clinical net benefit of different interventions. Finally, this study utilized change-from-baseline values and post-intervention measurements when pooling effect sizes, and follow-up durations varied significantly across studies (ranging from immediate assessment to 12 months). Although the random-effects model partially accounted for inter-study heterogeneity, the potential impact of different follow-up durations and baseline levels on MD comparability remained unresolved. Nevertheless, the robustness of the study conclusions was not undermined by these limitations, and the scope of application and evidence strength were clarified. The core value of this study lies in systematically synthesizing available evidence to identify relative advantages of different interventions in terms of outcome metrics. This guided the direction of future head-to-head comparative RCTs, long-follow-up designs, standardized outcome reporting, and scientific research. Future high-quality RCTs with larger samples and longer follow-up can focus on comparing VR with traditional training, the dose-response relation of RT, and the individualized application of combination training across populations with different fracture risks.

## 5 Conclusion

This study systematically compared the relative effects of different exercise regimens on older OP patients. The results suggested that the benefits of exercise therapies were protocol-specific. VR might enhance balance, RT might offer advantages in reducing fall risk, and BT + RT + aerobics exhibited potential benefits in improving BMD. However, given the limited number of included studies, sparse network structures, and overlapping CIs of some interventions, current findings should be treated cautiously. This study also had limitations: Due to short follow-up periods, inferences about long-term efficacy, sustainability, and safety of interventions were precluded. Some studies had RoB due to small sample sizes and inadequate blinding. No quantitative analyses were conducted on key indicators for scientific implementation, such as compliance, adverse events, and economic feasibility. Therefore, our findings were only a relative comparison based on available evidence and were insufficient to serve as a basis for developing specific clinical pathways. Future rigorously designed high-quality RCTs with large sample sizes and longer follow-up are needed to further validate the long-term efficacy and safety of different interventions, and identify appropriate populations. Moreover, key indicators for scientific implementation, such as compliance and cost-effectiveness, can be deeply explored to facilitate the translation of interventions from evidence to clinical practice.

## Supplementary material

Supplementary Figure 1 League table for BBS scores.

Supplementary Figure 2 Inconsistency test for BBS scores.

Supplementary Figure 3 League table for TUG.

Supplementary Figure 4 Inconsistency test for TUG.

Supplementary Figure 5 League table for BMD.

Supplementary Figure 6 League table for OLS.

Supplementary Figure 7 League table for falls.

Supplementary Table 1 Node-splitting analysis results for the BBS network.

Supplementary Table 2 Node-splitting analysis results for the TUG network.
