# Correction: Effects of exercise regimens on balance ability in older patients with osteoporosis: a systematic review and Bayesian network meta-analysis of randomized controlled trials

**DOI:** 10.3389/fphys.2026.1853138

**Published:** 2026-04-29

**Authors:** Xiangyue Liu, Mengjing Chang, Huiyun Yuan, Xuemei Zheng, Wenling Tian, Dongwen Li, Dongfa Liao, Lin Cui

**Affiliations:** 1 College of Nursing, Chengdu Medical College, Chengdu, Sichuan, China; 2 Orthopaedics Department, The General Hospital of the Western Theater Command of the People’s Liberation Army, Chengdu, Sichuan, China; 3 College of Nursing, North Sichuan Medical College, Nanchong, Sichuan, China; 4 Stomatology Department, The General Hospital of the Western Theater Command of the People’s Liberation Army, Chengdu, Sichuan, China; 5 Nursing Department, The General Hospital of the Western Theater Command of the People’s Liberation Army, Chengdu, Sichuan, China

**Keywords:** balance, exercise regimen, network meta-analysis, older, osteoporosis

Affiliation “College of Nursing, North Sichuan Medical College, Nanchong, Sichuan, China” was erroneously given as “College of Nursing, Chengdu Medical College, Chengdu, Sichuan, China” for authors Xuemei Zheng (4th author) and Wenling Tian (5th author).

The original version of this article has been updated.

