# Supplementary figures and images for: Effects of exercise regimens on balance ability in older patients with osteoporosis: a systematic review and Bayesian network meta-analysis of randomized controlled trials
Source: Front Physiol. 2026 Mar 31;17:1793389. doi: 10.3389/fphys.2026.1793389 (PMC13076118; doi:10.3389/fphys.2026.1793389)

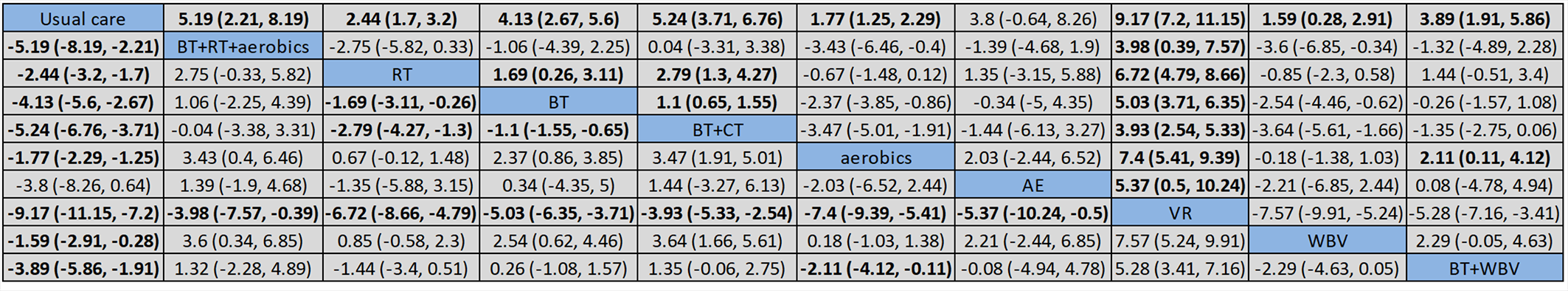

Supplement: Supplementary Figure 1 — League table for BBS scores. [file Image1.tif]

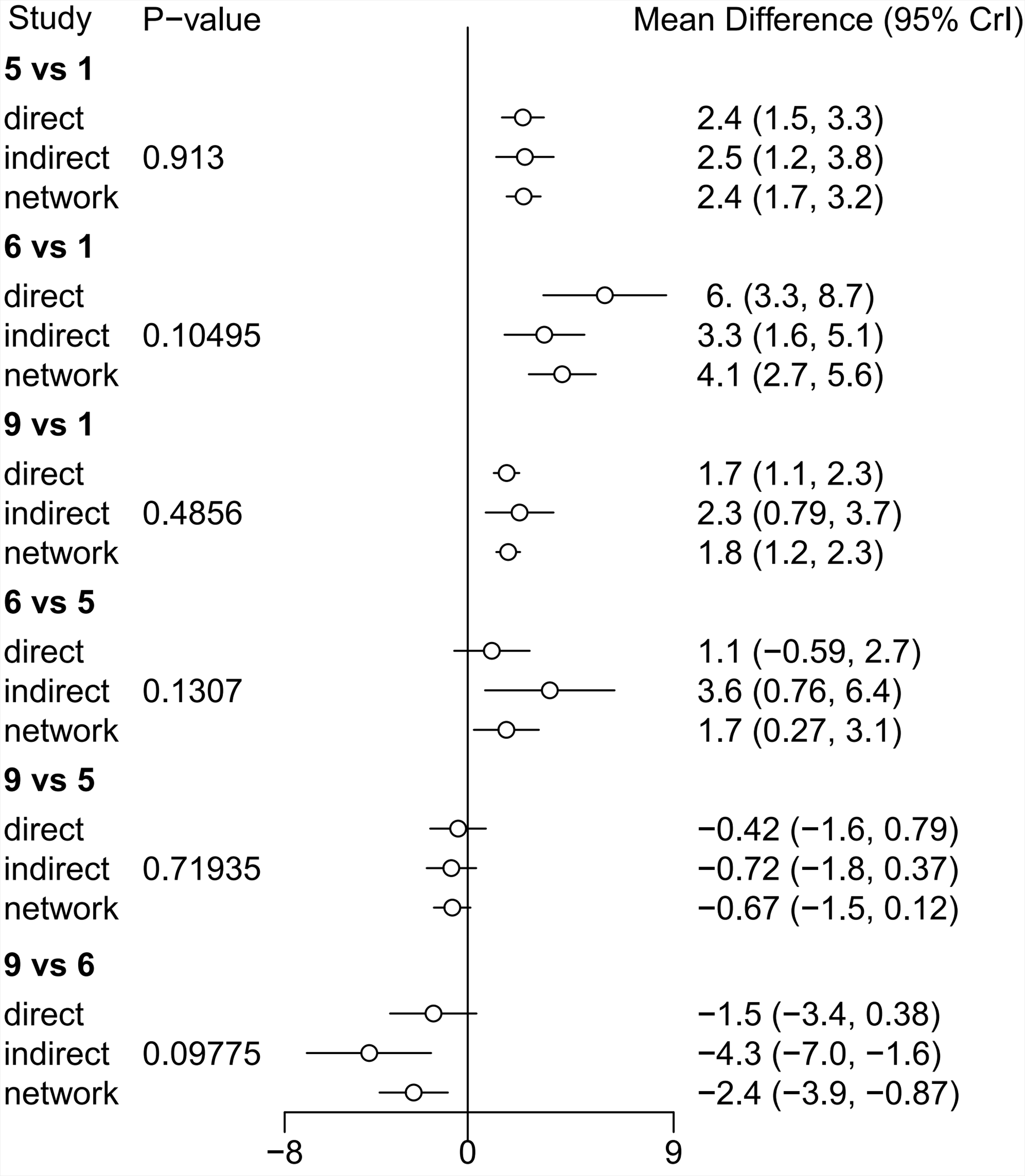

Supplement: Supplementary Figure 2 — Inconsistency test for BBS scores. [file Image2.tif]

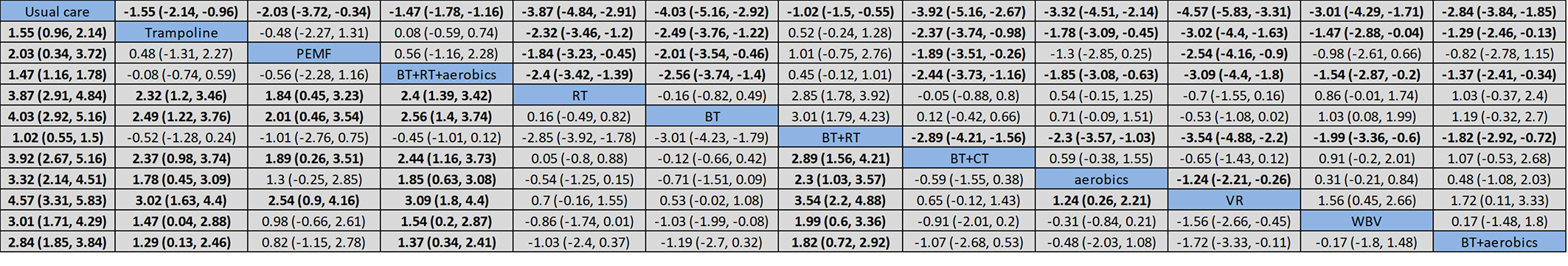

Supplement: Supplementary Figure 3 — League table for TUG. [file Image3.tif]

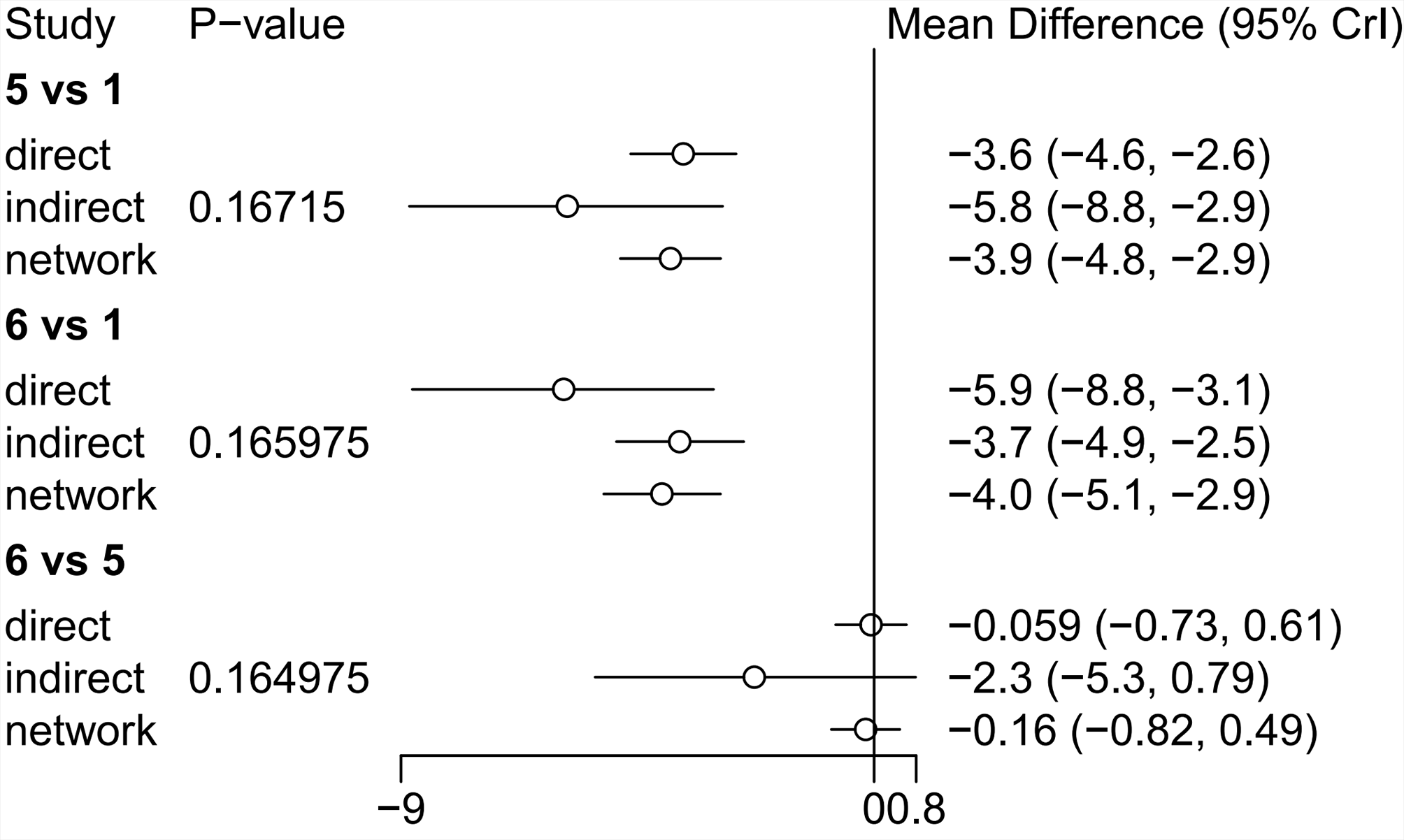

Supplement: Supplementary Figure 4 — Inconsistency test for TUG. [file Image4.tif]

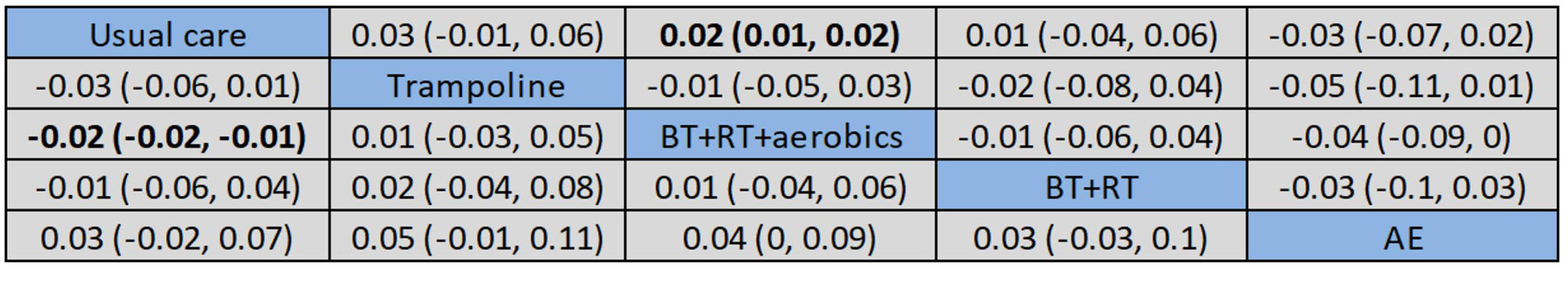

Supplement: Supplementary Figure 5 — League table for BMD. [file Image5.tif]

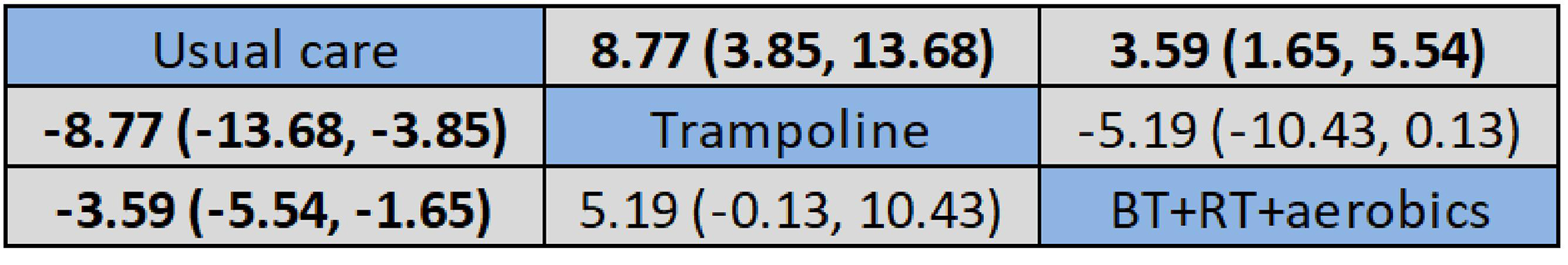

Supplement: Supplementary Figure 6 — League table for OLS. [file Image6.tif]

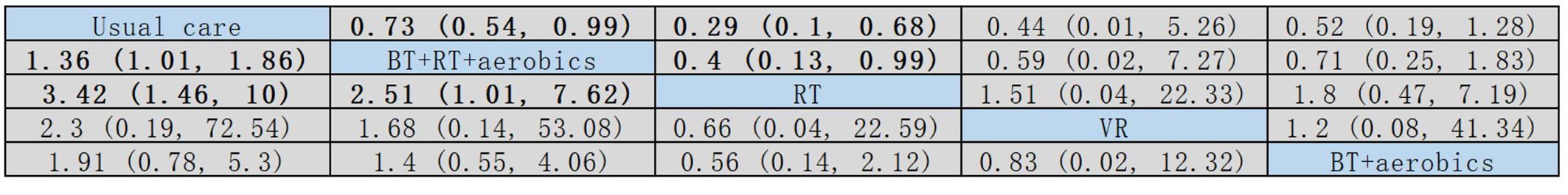

Supplement: Supplementary Figure 7 — League table for falls. [file Image7.tif]
