# Supplementary material for: Effects of exercise regimens on balance ability in older patients with osteoporosis: a systematic review and Bayesian network meta-analysis of randomized controlled trials
Source: Front Physiol. 2026 Mar 31;17:1793389. doi: 10.3389/fphys.2026.1793389 (PMC13076118; doi:10.3389/fphys.2026.1793389)
Supplement: Supplementary Table 1 — Node-splitting analysis results for the BBS network. [file Table1.docx]

Table S1 Node-splitting analysis results for the BBS network

| Study | Direct  MD (95% Crl ) | Indirect  MD (95% Crl ) | Network  MD (95% Crl ) | P-value |
| --- | --- | --- | --- | --- |
| RT vs Usual care | 2.4 (1.5, 3.3) | 2.5 (1.2, 3.8) | 2.4 (1.7, 3.2) | 0.913 |
| BT vs Usual care | 6.0 (3.3, 8.7) | 3.3 (1.6, 5.1) | 4.1 (2.7, 5.6) | 0.10495 |
| aerobics vs Usual care | 1.7 (1.1, 2.3) | 2.3 (0.79, 3.7) | 1.8 (1.2, 2.3) | 0.4865 |
| BT vs RT | 1.1 (-0.59, 2.7) | 3.6 (0.76, 6.4) | 1.7 (0.27, 3.1) | 0.1307 |
| aerobics vs RT | -0.42 (-1.6, 0.79) | -0.72 (-1.8, 0.37) | -0.67 (-1.5, 0.12) | 0.71935 |
| aerobics vs BT | -1.5 (-3.4, 0.38) | -4.3 (-7.0, -1.6) | -2.4 (-3.9, -0.87) | 0.09775 |

*Table note: MD: mean difference; RT: Resistance training; BT: Balance training.

Table S2 Node-splitting analysis results for the TUG network

| Study | Direct  MD (95% Crl ) | Indirect  MD (95% Crl ) | Network  MD (95% Crl ) | P-value |
| --- | --- | --- | --- | --- |
| RT vs Usual care | -3.6 (-4.6, -2.6) | -5.8 (-8.8, -2.9) | -3.9 (-4.8, -2.9) | 0.16715 |
| BT vs Usual care | -5.9 (-8.8, -3.1) | -3.7 (-4.9, -2.5) | -4.0 (-5.1, -2.9) | 0.165975 |
| BT vs RT | -0.059 (-0.73, 0.61) | -2.3 (-5.3, 0.79) | -0.16 (-0.82, 0.49) | 0.164975 |

*Table note: MD: mean difference; RT: Resistance training; BT: Balance training.
