# Supplementary material for: Effects of exercise regimens on balance ability in older patients with osteoporosis: a systematic review and Bayesian network meta-analysis of randomized controlled trials
Source: Front Physiol. 2026 Mar 31;17:1793389. doi: 10.3389/fphys.2026.1793389 (PMC13076118; doi:10.3389/fphys.2026.1793389)
Supplement: Supplementary file 9 [file DataSheet1.docx]

**Appendix 1 Search Results Record**

| Pubmed | | |
| --- | --- | --- |
| # | Query | Results |
| 1 | "Osteoporosis"[Mesh] | 67834 |
| 2 | Age Related Osteoporosis[Title/Abstract] OR Age-Related Bone Losses[Title/Abstract] OR Age-Related Osteoporoses[Title/Abstract] OR endocrine osteoporosis[Title/Abstract] OR Osteoporoses[Title/Abstract] OR osteoporotic decalcification[Title/Abstract] OR pathologic decalcification[Title/Abstract] OR Post-Traumatic Osteoporoses[Title/Abstract] OR Senile Osteoporoses[Title/Abstract] OR osteoporosis[Title/Abstract] | 95450 |
| 3 | #1 OR #2 | 113599 |
| 4 | "Exercise"[Mesh] | 275836 |
| 5 | Acute Exercises[Title/Abstract] OR Aerobic Exercises[Title/Abstract] OR biometric exercise[Title/Abstract] OR effort[Title/Abstract] OR exercise capacity[Title/Abstract] OR exercise performance[Title/Abstract] OR Exercise Trainings[Title/Abstract] OR Exercises[Title/Abstract] OR exertion[Title/Abstract] OR fitness training[Title/Abstract] OR fitness workout[Title/Abstract] OR Isometric Exercises[Title/Abstract] OR Physical Activities[Title/Abstract] OR physical effort[Title/Abstract] OR Physical Exercises[Title/Abstract] OR physical exertion[Title/Abstract] OR physical workout[Title/Abstract] OR physical work-out[Title/Abstract] OR exercise[Title/Abstract] | 590413 |
| 6 | #4 OR #5 | 729574 |
| 7 | "Postural Balance"[Mesh] | 31263 |
| 8 | body equilibrium[Title/Abstract] OR body sway[Title/Abstract] OR musculoskeletal equilibrium[Title/Abstract] OR postural balance[Title/Abstract] OR Postural Controls[Title/Abstract] OR postural equilibrium[Title/Abstract] OR Posture Balances[Title/Abstract] OR Posture Controls[Title/Abstract] OR Posture Equilibriums[Title/Abstract] OR balance[Title/Abstract] | 329203 |
| 9 | #7 OR #8 | 341223 |
| 10 | #3 AND #6 AND #9 | 531 |

| Embase | | |
| --- | --- | --- |
| # | Query | Results |
| 1 | 'osteoporosis'/exp | 176921 |
| 2 | 'age related osteoporosis':ab,ti OR 'age-related bone losses':ab,ti OR 'age-related osteoporoses':ab,ti OR 'endocrine osteoporosis':ab,ti OR osteoporoses:ab,ti OR 'osteoporotic decalcification':ab,ti OR 'pathologic decalcification':ab,ti OR 'post-traumatic osteoporoses':ab,ti OR 'senile osteoporoses':ab,ti OR osteoporosis:ab,ti | 140479 |
| 3 | #1 OR #2 | 203095 |
| 4 | 'exercise'/exp | 545746 |
| 5 | 'acute exercises':ab,ti OR 'aerobic exercises':ab,ti OR 'biometric exercise':ab,ti OR effort:ab,ti OR 'exercise capacity':ab,ti OR 'exercise performance':ab,ti OR 'exercise trainings':ab,ti OR exercises:ab,ti OR exertion:ab,ti OR 'fitness training':ab,ti OR 'fitness workout':ab,ti OR 'isometric exercises':ab,ti OR 'physical activities':ab,ti OR 'physical effort':ab,ti OR 'physical exercises':ab,ti OR 'physical exertion':ab,ti OR 'physical workout':ab,ti OR 'physical work-out':ab,ti OR exercise:ab,ti | 803762 |
| 6 | #4 OR #5 | 974465 |
| 7 | 'body equilibrium'/exp | 27746 |
| 8 | 'body equilibrium':ab,ti OR 'body sway':ab,ti OR 'musculoskeletal equilibrium':ab,ti OR 'postural balance':ab,ti OR 'postural controls':ab,ti OR 'postural equilibrium':ab,ti OR 'posture balances':ab,ti OR 'posture controls':ab,ti OR 'posture equilibriums':ab,ti OR balance:ab,ti | 416633 |
| 9 | #7 OR #8 | 425622 |
| 10 | #3 AND #6 AND #9 | 1062 |

| Cochrane Library | | |
| --- | --- | --- |
| # | Query | Results |
| 1 | MeSH descriptor: [Osteoporosis] explode all trees | 5537 |
| 2 | (Age Related Osteoporosis OR Age-Related Bone Losses OR Age-Related Osteoporoses OR endocrine osteoporosis OR Osteoporoses OR osteoporotic decalcification OR pathologic decalcification OR Post-Traumatic Osteoporoses OR Senile Osteoporoses OR osteoporosis):ti,ab,kw | 12826 |
| 3 | #1 or #2 | 12826 |
| 4 | MeSH descriptor: [Exercise] explode all trees | 40662 |
| 5 | (Acute Exercises OR Aerobic Exercises OR biometric exercise OR effort OR exercise capacity OR exercise performance OR Exercise Trainings OR Exercises OR exertion OR fitness training OR fitness workout OR Isometric Exercises OR Physical Activities OR physical effort OR Physical Exercises OR physical exertion OR physical workout OR physical work-out OR exercise):ti,ab,kw | 181421 |
| 6 | #4 or #5 | 185551 |
| 7 | MeSH descriptor: [Postural Balance] explode all trees | 4596 |
| 8 | (body equilibrium OR body sway OR musculoskeletal equilibrium OR postural balance OR Postural Controls OR postural equilibrium OR Posture Balances OR Posture Controls OR Posture Equilibriums OR balance):ti,ab,kw | 42339 |
| 9 | #7 OR #8 | 42352 |
| 10 | #3 AND #6 AND #9 | 400 |

| Web of science | | |
| --- | --- | --- |
| # | Query | Results |
| 1 | Age Related Osteoporosis OR Age-Related Bone Losses OR Age-Related Osteoporoses OR endocrine osteoporosis OR Osteoporoses OR osteoporotic decalcification OR pathologic decalcification OR Post-Traumatic Osteoporoses OR Senile Osteoporoses OR osteoporosis (Topic) and Preprint Citation Index (Exclude – Database) | 210682 |
| 2 | Acute Exercises OR Aerobic Exercises OR biometric exercise OR effort OR exercise capacity OR exercise performance OR Exercise Trainings OR Exercises OR exertion OR fitness training OR fitness workout OR Isometric Exercises OR Physical Activities OR physical effort OR Physical Exercises OR physical exertion OR physical workout OR physical work-out OR exercise (Topic) and Preprint Citation Index (Exclude – Database) | 3985938 |
| 3 | body equilibrium OR body sway OR musculoskeletal equilibrium OR postural balance OR Postural Controls OR postural equilibrium OR Posture Balances OR Posture Controls OR Posture Equilibriums OR balance (Topic) and Preprint Citation Index (Exclude – Database) | 1846331 |
| 4 | #3 AND #2 AND #1 and Preprint Citation Index (Exclude – Database) | 1807 |
